# Using newly optimized genetic tools to probe *Strongyloides* sensory behaviors

**DOI:** 10.1016/j.molbiopara.2022.111491

**Published:** 2022-07

**Authors:** Patricia Mendez, Breanna Walsh, Elissa A. Hallem

**Affiliations:** a Department of Microbiology, Immunology, and Molecular Genetics, University of California, Los Angeles, Los Angeles, CA, USA; b Molecular Biology Interdepartmental PhD Program, University of California Los Angeles, Los Angeles, CA, USA; c Medical Scientist Training Program, University of California, Los Angeles, Los Angeles, CA, USA; d Molecular Biology Institute, University of California, Los Angeles, Los Angeles, CA, USA

**Keywords:** Parasitic nematodes, *Strongyloides*, Chemosensation, Thermosensation, Sensory behavior, Host-seeking behavior

## Abstract

The oft-neglected human-parasitic threadworm, *Strongyloides stercoralis*, infects roughly eight percent of the global population, placing disproportionate medical and economic burden upon marginalized communities. While current chemotherapies treat strongyloidiasis, disease recrudescence and the looming threat of anthelminthic resistance necessitate novel strategies for nematode control. Throughout its life cycle, *S. stercoralis* relies upon sensory cues to aid in environmental navigation and coordinate developmental progression. Odorants, tastants, gases, and temperature have been shown to shape parasite behaviors that drive host seeking and infectivity; however, many of these sensory behaviors remain poorly understood, and their underlying molecular and neural mechanisms are largely uncharacterized. Disruption of sensory circuits essential to parasitism presents a promising strategy for future interventions. In this review, we describe our current understanding of sensory behaviors – namely olfactory, gustatory, gas sensing, and thermosensory behaviors – in *Strongyloides* spp. We also highlight the ever-growing cache of genetic tools optimized for use in *Strongyloides* that have facilitated these findings, including transgenesis, CRISPR/Cas9-mediated mutagenesis, RNAi, chemogenetic neuronal silencing, and the use of fluorescent biosensors to measure neuronal activity. Bolstered by these tools, we are poised to enter an era of rapid discovery in *Strongyloides* sensory neurobiology, which has the potential to shape pioneering advances in the prevention and treatment of strongyloidiasis.

## Highlights

• Transgenesis, CRISPR, and RNAi have been successfully applied to *Strongyloides* spp.
• This genetic toolkit is transforming the study of *Strongyloides* sensory biology.
• *Strongyloides* infective larvae host seek using host-emitted sensory cues.
• CRISPR mutagenesis identified a cGMP pathway required for host seeking.
• Calcium imaging identified functional adaptations of *Strongyloides* sensory neurons.

## 1 Introduction

*Strongyloides stercoralis* is a soil-transmitted parasitic nematode that infects humans via direct skin penetration and causes the clinical disease strongyloidiasis [1]. In 2017, the global prevalence of strongyloidiasis was estimated at 600 million individuals, with over three-quarters of these cases located in Southeast Asia, Africa, and the Western Pacific [2]. The prevalence of strongyloidiasis is likely underestimated, given that half of cases are asymptomatic and traditional stool-based microscopy detection methods have only limited specificity and sensitivity [3], [4].

While classified as a neglected tropical disease, strongyloidiasis has been better described as a disease of disadvantage, predominantly impacting communities with insufficient access to sanitation infrastructure [5], [6]. For example, throughout the United States, cases of strongyloidiasis have been detected in rural, socioeconomically depressed, and marginalized communities, despite the country’s location beyond the tropics [5], [7], [8], [9], [10], [11], [12]. Globally, populations at especially high risk of strongyloidiasis include military veterans, immigrants and travelers from endemic areas, immunocompromised individuals, members of indigenous communities, and those with occupational exposure to soil (e.g.*,* farmers and coal miners) [5], [13], [14], [15], [16], [17], [18]. The host range of *S. stercoralis* includes humans, non-human primates, dogs, and cats; zoonotic infections have been described and dogs are suggested to be an important reservoir of disease [19], [20], [21], [22], [23].

**Fig. 1:**
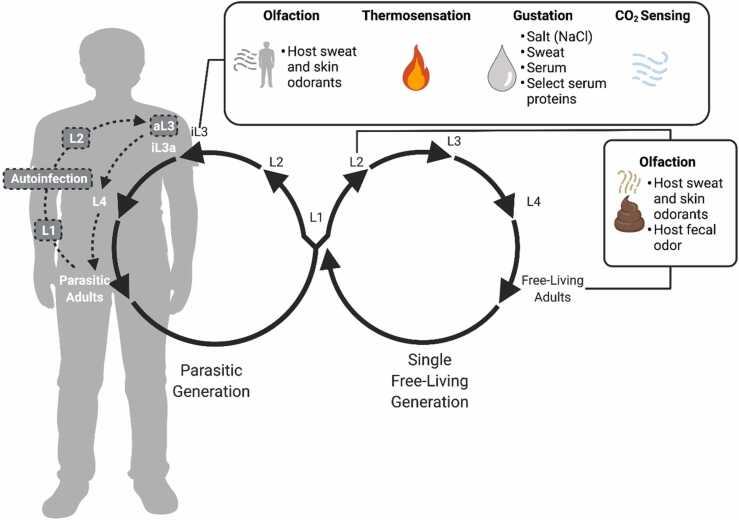
The role of sensory cues in the *S. stercoralis* life cycle. The life cycle of *S. stercoralis* consists of a parasitic generation and a single free-living generation [24]. Infective third-stage larvae (iL3s) navigate through the soil in search of a host. Host seeking involves attraction to host-emitted olfactory, thermosensory, and gustatory cues, as well as repulsion from carbon dioxide [27], [28], [29], [30], [31], [32], [33], [34], [35] (top box). Upon entry into a host via skin penetration, the developmentally arrested iL3s resume development via a process called activation. Activated iL3s (iL3as) develop into fourth-stage larvae (L4s) and progress to parasitic adulthood, where they reproduce by parthenogenesis in the small intestine of the host. The progeny then exit the host via excretion as first-stage larvae (L1s) or develop into autoinfective third-stage larvae (aL3s) within the host large intestine and then reinfect the same host in a process called autoinfection. The L1 progeny that exit the host may enter either a homogonic or heterogonic life cycle. The homogonic generation develops into iL3s that find and infect a new host. The heterogonic generation progresses through the L1-L4 stages and develops into free-living adults that yield progeny via sexual reproduction. *S. stercoralis* has a single free-living generation; all progeny of free-living adults develop into iL3s [24]. The free-living adults are attracted to both host skin and sweat odorants, and host fecal odor and its constituent odorants [27], [34] (right box). Attraction to fecal odor likely serves to maintain the free-living adults on host feces, where they grow and reproduce. Mixed-stage free-living larval populations (comprised primarily of post-parasitic L2s) are also attracted to fecal odor [27]. The behavioral responses of intra-host life stages, including iL3as and parasitic adults, remain to be investigated. Life stages and processes in white text within the human silhouette denote portions of the *S. stercoralis* life cycle exclusively within a host, while stages in black text denote stages found in the external environment.

The life cycle of *S. stercoralis* is unique among human-parasitic nematodes, featuring a single environmentally free-living generation and a clinically relevant autoinfective cycle [24], [25] (Fig. 1). After developmentally arrested infective third-stage larvae (iL3s) breach the host skin barrier, the nematodes resume developmental progression via a process called activation. The larvae undergo intra-host navigation, and ultimately home toward the host intestinal tract. Many reports suggest that worms first traverse the vasculature to the pulmonary alveoli and migrate up the respiratory tree, at which point they are propelled into the pharynx via cough and subsequently swallowed into the digestive tract [25]. However, there is also evidence suggesting that worms can migrate directly to the host intestine [26]. Within the small intestine, parasitic female adults perform parthenogenesis and generate clonal progeny that are subject to three distinct fates: 1) an autoinfective homogonic cycle, 2) a free-living homogonic cycle, and 3) a free-living heterogonic cycle (Fig. 1). In the autoinfective homogonic cycle, post-parasitic larvae develop into autoinfective third-stage larvae (aL3s) within the host and reinfect via penetration of large intestinal tissues or the perianal skin. In contrast, post-parasitic larvae destined for the homogonic and heterogonic free-living cycles exit the host in feces. Within the environment, homogonic progeny develop directly into iL3s and seek a tenable host. Heterogonic progeny develop into free-living male and female adults that use sexual reproduction to yield post-free-living larvae. These larvae then mature to iL3s and pursue a new host [25].

While many strongyloidiasis cases are asymptomatic, symptomatic patients can experience a range of dermatologic, pulmonary, and gastrointestinal sequelae [36]. Because of the nematode’s unique capacity for autoinfection, individuals with indolent, undiagnosed strongyloidiasis are at risk for hyperinfection syndrome and disseminated disease upon iatrogenic or illness-induced immune suppression [37]. Disseminated strongyloidiasis ─ wherein larvae invade numerous tissues such as heart, liver, and brain [38] ─ is fatal in most cases [39]. Individuals co-infected with human T-cell lymphotropic virus type 1 (HTLV-1), suffering from hematologic malignancy, undergoing solid organ transplant, or receiving corticosteroid treatments are at particular risk of hyperinfection syndrome and disseminated strongyloidiasis [40], [41]. Recent case studies demonstrate that treatment of SARS-CoV-2 infection with corticosteroids and immunosuppressive agents can induce symptomatic strongyloidiasis, indicative of hyperinfection syndrome [42], [43], [44]. A single dose of ivermectin is the gold standard of treatment for uncomplicated strongyloidiasis in most patients, while a four-dose regimen is recommended for immunocompromised individuals [45], [46]. Albendazole is also effective in treating strongyloidiasis and is used in settings where ivermectin is not readily available [1], [47], [48]. Most patients are clinically cured; however, if even a single worm survives following treatment, the autoinfective cycle can mediate recrudescent infection [49]. Despite the high cure rate in patients treated with anthelminthics, these medications do not prevent reinfection [50]. Moreover, the emergence of anthelminthic resistance in human-infective parasitic worms is of growing concern, as drug-resistant nematodes of livestock are already pervasive [51], [52], [53], [54], [55].

Strongyloidiasis is often unrecognized and misdiagnosed by clinicians outside of endemic areas [56], [57]. This poses a challenge to clinical management, as the geographic range of soil-transmitted helminths is expected to expand with climate change [58], [59]. *S. stercoralis* is well-suited to warm, humid environments wherein free-living iL3s can survive in soil for weeks before finding a new host [2], [60]. Already, autochthonous cases of strongyloidiasis have been detected in regions considered temperate [23], [61], [62]. Large weather events and natural disasters have the potential to disrupt fragile sanitation infrastructure [63], while also displacing individuals as climate refugees [64]. For decades, refugees have been at particularly high risk for strongyloidiasis, reflecting the lack of adequate sanitation and high population density of temporary shelters [65], [66].

Acknowledging these challenges to the eradication of strongyloidiasis, novel interventions aimed to disrupt the parasite’s infective cycle are urgently needed. Targets for such interventions are likely to be illuminated through increased understanding of the environmental cues that dictate parasitic nematode behavior. To date, environmental signals sensed by *S. stercoralis* include odorants, tastants, gases, and heat. These signals, via largely uncharacterized neural circuits and molecular pathways, direct host-seeking behaviors in iL3s and impact developmental progression through the life cycle (Fig. 1). Exogenous cues are also suggested to impact larval navigation within the host and augment parasite tropism to specific host tissues. The neural basis of these sensory behaviors is an area ripe for active research. The nervous system of *S. stercoralis* is a well-suited target for future chemotherapeutic agents and environmental controls [67].

Buoyed by experimentally manipulable free-living life cycle stages, technical advances have poised the field toward a renaissance in the study of *S. stercoralis* biology. A rich area of current *Strongyloides* research centers on the neural basis of sensory behavior. Here, we describe genetic techniques demonstrated as tractable in *S. stercoralis* and the closely related rat parasite *Strongyloides ratti*, including transgenesis and Clustered Regularly Interspaced Short Palindromic Repeats (CRISPR)/Cas9-mediated mutagenesis. We then discuss how these tools, in combination with rigorous behavioral assays, are being leveraged to understand parasite gustation, olfaction, gas sensation, and thermosensation. Chemogenetic neuronal silencing and the use of genetically encoded fluorescent biosensors ─ neuroscience techniques recently optimized for use in *Strongyloides* ─ have allowed for novel interrogation of neuron function underlying sensory behavior. Indeed, this arsenal of new, robust experimental techniques is enabling mechanistic analyses of sensory behaviors in this medically and economically important parasitic nematode.

### 1.1 *S. stercoralis* and *S. ratti* are genetically tractable model systems for the study of sensory behavior

**Fig. 2:**
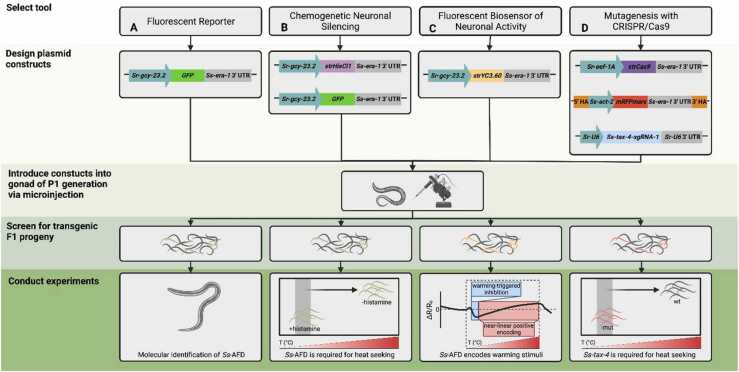
Genetic tools optimized for use in *Strongyloides* have illuminated the molecular and neural basis of heat-seeking behavior. These genetic tools each rely upon intragonadal microinjection of *Strongyloides* free-living adult females. **A.** Fluorescent reporters allow for molecular identification of neurons. In this example, the *Sr-gcy-23.2* promoter drives expression of *GFP* in a pair of head neurons, allowing for selection of transgenic progeny. Morphologic and anatomic identification, in combination with molecular identification, aid characterization of the GFP-positive neurons, previously called the *Ss*-ALD neurons [35], [69], as the *Strongyloides* homologs of the *C. elegans* AFD neurons [29]. Thus, these neurons are hereafter referred to as the *Ss*-AFD neurons [29]. **B.** Chemogenetic silencing is achieved by expression of the histamine-gated chloride channel HisCl1 from a neuron-specific promoter. When transgenic worms are treated with exogenous histamine, the resultant influx of chloride silences the neuron of interest. In this example, the *Sr-gcy-23.2* promoter drives expression of a codon-optimized gene encoding HisCl1 specifically in *Ss*-AFD, thereby silencing *Ss*-AFD. Co-injection of a transgene encoding GFP driven by the same promoter provides a selectable marker of transgenesis. Chemogenetic silencing of *Ss*-AFD disrupts heat-seeking behavior in *S. stercoralis* iL3s. **C.** Extrachromosomal expression of a genetically encoded calcium indicator enables recording of neuronal activity. Expression of a codon-optimized gene encoding yellow cameleon YC3.60 was driven by the *Sr*-*gcy-23.2* promoter, enabling imaging of the *Ss*-AFD neuron. Calcium schematic shows an initial hyperpolarization followed by a near-linear, positive encoding of temperature change spanning a range from ambient to host body temperature. **D.** Gene knockouts are feasible with CRISPR/Cas9-mediated mutagenesis. This approach uses three plasmids: 1) a plasmid containing a codon-optimized *Cas9* gene, 2) a plasmid encoding a single guide RNA (sgRNA) cassette specific to the gene of interest, and 3) a homology-directed repair template that introduces *mRFPmars* driven by the *Ss*-*act-2* promoter into the disrupted gene of interest. iL3s that express mRFPmars in body wall muscle are selected for experimental study, and genotyped post hoc. In this example, the *Ss-act-2p::mRFPmars::Ss-era-1* 3’UTR cassette is introduced into the *Ss-tax-4* locus, abolishing expression of *Ss-tax-4*. *Ss-tax-4* homozygous mutant iL3s are unable to heat seek, demonstrating that *Ss-tax-4* is critical to temperature sensation. Schematics in the bottom row are based on the data from Bryant et al., 2018 [28] and Bryant et al.*,* 2022 [29]. “wt” = wild-type iL3s; “mut” = *Ss*-*tax-4* homozygous knockout iL3s; “HA” = homology arm.

In parasitic nematodes, the historic lack of genetic and genomic tools has proved a barrier to elucidating the molecular mechanisms of host-parasite interactions. As a result, efforts to identify molecular targets for vaccine development, prophylaxis, and clinical treatment have been hampered. However, recent advances in the application of genetic and genomic tools to *Strongyloides* spp. have positioned the genus as a genetic model system for mechanistic studies of parasitic nematode biology. *Strongyloides* spp. are now amenable to techniques for studying gene function such as transgenesis, RNA interference (RNAi), and CRISPR/Cas9-mediated mutagenesis [68]. Genetically encoded tools for the precise study of neuronal function have also recently been optimized; these techniques include chemogenetic neuronal silencing and the use of fluorescent biosensors to monitor neuronal activity [29] (Fig. 2). With these techniques in hand, mechanistic exploration of sensory pathways and sensory circuits in *Strongyloides* is increasingly feasible.

The genomic and transcriptomic sequencing of *Strongyloides* spp. has catalyzed developments in the genetic manipulation of this genus. For *S. stercoralis*, *S. ratti, Strongyloides venezuelensis,* and *Strongyloides papillosus,* genomic sequences and life-stage-specific RNA sequencing (RNA-seq) data are available [70], [71], [72], [73], [74]. Furthermore, online resources such as WormBase ParaSite and tools such as the *Strongyloides* RNA-seq Browser, the Wild Worm Codon Adapter, and the Nematode Chemoreceptor Database (NemChR-DB) have facilitated efforts to identify homologous genes across nematode species and to study the functions of these genes in *Strongyloides* spp. [73], [75], [76], [77].

As genetic model organisms for other parasitic nematodes, *Strongyloides* spp. offer the advantage of an environmental, free-living adult life stage, which grants the opportunity to adapt genetic tools developed for the free-living nematode *Caenorhabditis elegans.* Thus far, nematodes of the *Strongyloides* and *Parastrongyloides* genera are the only parasitic nematodes known to have at least one free-living adult life stage outside their hosts [78], [79], [80]. Anatomical similarities between *Strongyloides* free-living adult females and *C. elegans* adult hermaphrodites made it possible to adopt the *C. elegans* technique for intragonadal microinjection of exogenous DNA in *Strongyloides* [68], [81], [82], [83], [84], [85], [86], [87]. In *Strongyloides*, intragonadal microinjection of exogenous DNA is also possible in free-living males [88].

### 1.2 Transgenesis in Strongyloides species

To generate transgenic animals, the transgene of interest ─ flanked by a *Strongyloides* tissue-specific promoter and a *Strongyloides* 3’ untranslated region (UTR) ─ is introduced into a free-living adult by intragonadal microinjection [81], [82], [83], [84], [85], [86]. The resultant progeny (the F_1_ generation) express the transgene of interest from an extrachromosomal array [82], [83], [84], [85], [86]. In *Strongyloides* spp., extrachromosomal arrays are silenced after the F_1_ generation; thus, genomic integration is required for stable transgene expression in subsequent generations (F_2_, F_3_, etc.) [82], [83], [84]. Stable, heritable transgene expression in *S. ratti* has been successfully established with the use of a piggyBac transposase system for integration [89], [90]. The piggyBac system has also been used to generate *S. stercoralis* F_2_ iL3s expressing a transgene of interest [79].

The establishment and maintenance of stable transgenic lines in *Strongyloides* spp. requires passaging transgenic iL3s through a host, as the endoparasitic life cycle stages cannot yet be supported by in vitro culture [68]. As a result, generating and maintaining stable lines of *S. stercoralis* is often unattainable, due to both the relatively low efficiency of transgenesis and the prohibitively large number of iL3s with genome-integrated transgenes needed to establish a patent infection in a laboratory host. Current laboratory hosts for *S. stercoralis* – dogs and gerbils – require substantial numbers of transgenic iL3s to initiate and maintain a successful infection. In contrast, establishing stable lines of *S. ratti* is feasible because only a few transgenic iL3s are required to establish an infection in its natural host, the rat [91]. Thus, if a stable line is needed ─ either for assays that require large numbers of transgenic iL3s or for assays that require other transgenic life stages ─ *S. ratti* is likely to be the model organism of choice [68].

In the context of sensory behavior, the ability to generate transgenic iL3s has enabled the identification of genes that are expressed in sensory neurons, which are thought to play an important role in sensory function (Fig. 2A). For example, a reporter construct consisting of the promoter for the *S. stercoralis* homolog of the cyclic guanosine monophosphate (cGMP)-gated cation channel gene *tax-4* upstream of the gene encoding green fluorescent protein (GFP) was found to express in a subset of head sensory neurons [28], and was then demonstrated to be required for thermosensory and chemosensory behaviors [28], [29], [34]. More recently, transgenesis has been used for the chemogenetic silencing of neurons via cell-specific expression of the histamine-gated chloride channel HisCl1 (Fig. 2B), which reduces neuronal activity in the presence of exogenous histamine [29], [92]; the monitoring of calcium levels via the ratiometric calcium sensor yellow cameleon YC3.60, which serves as an indicator of neuronal activity [29], [93] (Fig. 2C); and the measurement of cGMP levels using the GFP-based cGMP biosensor FlincG3 [29], [94], [95]. Each of these transgenes was codon-optimized for use in *Strongyloides*, which improves expression [29], [77]. As in other organisms, P2A-mediated bicistronic plasmid vectors [96] can be used in *Strongyloides* to produce multiple distinct proteins from a single open reading frame [86]. The use of P2A-mediated bicistronic plasmid vectors is particularly useful for colocalization of non-fluorescent proteins (e.g.*,* HisCl1) with fluorescent proteins (e.g.*,* GFP), enabling efficient identification of transgenic progeny [29]. While these techniques are expected to be applicable to all or nearly all neuron types, a major limitation currently is the scarcity of identified neuron-specific promoters for *Strongyloides* spp. Identifying additional promoters that drive cell-specific expression in *Strongyloides* is thus a top priority.

### 1.3 CRISPR/Cas9-mediated targeted mutagenesis in Strongyloides species

The CRISPR/Cas9 system is a genome-editing tool that has been modified for use in a wide variety of organisms [97]. This system was recently adapted for use in *S. stercoralis* and *S. ratti*, enabling the first targeted gene disruptions in parasitic worms [68], [86], [98] (Fig. 2D). CRISPR/Cas9 has now been used successfully to disrupt several *Strongyloides* genes, including the FOXO transcription factor gene *Ss-daf-16* [86], the twitchin genes *Ss-unc-22* and *Sr-unc-22* [98], the collagen gene *Ss-rol-6* [99], the cGMP-gated cation channel subunit gene *Ss-tax-4* [28], [29], [34], [98], the nuclear hormone receptor gene *Ss*-*daf-12* [100], the DAF-12 co-regulator gene *Ss-dip-1* [100], and the cytochrome P450 gene *Ss-cyp22a9* [101].

To generate targeted gene knockouts in *S. stercoralis* and *S. ratti*, the CRISPR/Cas9 components can be introduced via DNA plasmids into free-living female adults by intragonadal microinjection, with mutagenesis occurring in the F_1_ progeny. The CRISPR/Cas9 components are delivered using three plasmids: 1) a plasmid encoding a single guide RNA (sgRNA); 2) a plasmid encoding the Cas9 endonuclease; and 3) a plasmid encoding a template for homology-directed repair (HDR) that contains a reporter cassette, such as *Ss-act-2p::mRFPmars::Ss-era-1* 3’UTR (Fig. 2D). The *Ss-act-2* promoter drives robust expression in body wall muscle, allowing candidate knockout F_1_ iL3s to be identified by the expression of mRFPmars along the length of the body wall. Importantly, only a subset of the F_1_ iL3s expressing the *Ss*-*act-2p::mRFPmars::Ss-era-1* 3’UTR reporter will have a homozygous gene disruption; some will have a heterozygous disruption, some will be mosaic, and some will exclusively express mRFPmars from an extrachromosomal array [68], [98]. Thus, individual mRFPmars-expressing iL3s must be subjected to behavioral or phenotypic analyses blind to genotype, and then PCR-genotyped post hoc to identify those that contain homozygous gene disruptions [68], [98]. To generate stable lines of knockout parasites, iL3s containing gene disruptions must be passaged through a host [68]. However, the ability to readily obtain homozygous knockouts in the F_1_ generation often alleviates the need for stable knockout lines and is especially critical for genes essential for host infection, wherein homozygous disruption precludes host passage.

### 1.4 RNAi in *Strongyloides* species

RNAi has also been successfully adapted for use in *S. ratti*. The successful application of RNAi to parasitic nematodes has been challenging, with variable efficacy across species and gene targets [102]. In the case of *S. ratti*, RNAi-enhancing genes, double-stranded DNA uptake-associated genes, and other RNAi-affiliated genes have not been identified [103]. To bypass these complications, a small interfering RNA soaking method devised in *Brugia malayi* has been adopted and utilized in *S. ratti* to knock down the phenotypically well-defined *daf-12* gene [103], [104]. Thus, RNAi provides a promising alternative to the CRISPR/Cas9 system for studying gene function and will be particularly useful in situations when large numbers of iL3s are required, genes of interest lack efficient Cas9 target sites, functional redundancy among genes necessitates the simultaneous knockdown of large numbers of genes, and/or complete knockout of a gene is lethal.

## 2 Conserved sensory neuroanatomy in *Strongyloides* species

For decades, the free-living nematode *C. elegans* has served as a powerful model in the study of sensory neurobiology. To probe the molecular and neural underpinnings of sensory behavior in *Strongyloides* spp. and other parasitic nematodes, insights gleaned from *C. elegans* provide a solid foundation for inquiry [105]. While *C. elegans* and *Strongyloides* spp. occupy discrete clades within the nematode phylogenetic tree [106], [107], [108], relative conservation in neuronal structure across nematode species allows for the identification of homologous sensory machinery [105].

In *C. elegans*, the anteriorly located, bilateral amphids serve as the primary sensory organs [109], [110]. Additional sensory organs include the phasmid and inner labial organs [109], [110]. The amphids are each composed of two glial cells (the socket cell and sheath cell) that encircle the ciliated dendrites of twelve sensory neurons [110]. These neurons are named for the morphology of their ciliated dendrites. For example, amphid finger neuron D (AFD) is the primary thermosensory neuron enclosed in the amphid, and features a dendritic tip laden with finger-like processes [111]. The dendritic tips of many of the sensory neurons are exposed to the external environment for the detection of sensory cues [109], [110]. Other sensory neurons, such as the CO_2_- and O_2_-sensing BAG neurons [112], [113] and the O_2_-sensing URX, AQR, and PQR neurons [113], [114], are not contained within sensory organs [110]. By electron microscopy, *S. stercoralis* was shown to have similar amphidal structures, albeit with thirteen putative sensory neurons enclosed [69], [115]. When comparing the *S. stercoralis* amphid to that of *C. elegans*, the anatomical position of the sensory neurons provides clues as to their functional identity. Moreover, laser ablation studies have verified that the sensory modalities of a subset of amphid neurons are conserved in *S. stercoralis* and *C. elegans* [35], [116], [117], [118], [119].

While homology crucially facilitates initial forays into understanding sensory behavior, divergent molecular sensors, neural encoding, and dendritic structures between *C. elegans* and *Strongyloides* spp. deliver exciting avenues for future research. These deviations suggest possible mechanisms by which *Strongyloides* spp. have adapted to parasitism [105]. Host seeking, host identification, skin penetration, and intra-host tissue tropism all likely depend upon co-opted sensory responses tailored to the invasive cycle.

## 3 Olfactory behaviors of *Strongyloides* species

*Strongyloides* species respond to a wide range of olfactory, or volatile chemical, cues. Given the narrow host ranges of most mammalian-parasitic nematodes, species-specific olfactory cues are likely critical for the ability of iL3s to distinguish hosts from non-hosts. Consistent with this possibility, *S. stercoralis* is attracted to multiple odorants emitted from human skin and sweat [27], [30], [34]. One such attractant is urocanic acid, an odorant largely specific to mammalian skin [30]. Interestingly, urocanic acid is found in the largest concentrations on the sole of the foot relative to other areas of the human body [120], consistent with the tendency of *S. stercoralis* to infect through the skin of the feet. Many of the odorants that are attractive to *S. stercoralis* are also attractive to anthropophilic mosquitoes, suggesting that parasitic worms and mosquitoes may target humans using many of the same olfactory cues [27].

A comparison of olfactory responses across parasitic nematode species revealed that some phylogenetically distant parasitic nematodes that share similar host ranges show overlapping olfactory preferences [27]. For instance, the two distantly related rat-infective parasites *S. ratti* and *Nippostrongylus brasiliensis* are attracted to a similar set of mammalian-emitted odorants, suggesting their olfactory behavior reflects their host preferences, rather than phylogeny [27]. However, *S. stercoralis* and the human-infective hookworm *Ancylostoma ceylanicum* have very different olfactory preferences, even though both species are skin-penetrating human parasites [34]. Notably, *S. stercoralis* is attracted primarily to skin and sweat odorants, whereas *A. ceylanicum* is attracted primarily to fecal odorants. This difference may reflect the fact that hookworms infect by skin penetration and fecal-oral transmission, whereas *S. stercoralis* infects primarily by skin penetration [34], [121], [122], [123].

Olfactory preferences vary across *Strongyloides* life cycle stages. In *S. ratti* and *S. stercoralis,* iL3s, free-living adults, and free-living larvae are attracted to host-emitted odorants found in skin and sweat, indicating that attraction to host odorants is not limited to infective life stages [27], [34] (Fig. 1). However, preferences for fecal odor vary across life stages such that free-living adults and non-infective larvae are attracted to host fecal odor and its constituent odorants, whereas iL3s are neutral to fecal odor [27], [34]. Preferences for bacteria also vary across life stages: *S. ratti* and *S. stercoralis* free-living adults are broadly attracted to host-associated and environmental bacteria, whereas the iL3s of both species are more narrowly attracted to a subset of environmental bacteria [124]. Moreover, the olfactory preferences of *S. stercoralis* iL3s more closely resemble those of *S. ratti* iL3s than those of *S. stercoralis* free-living adults [34]. Together, these findings suggest that differences in olfaction may play a role in life-stage-specific behaviors. Furthermore, these findings support a model in which decreased fecal attraction in iL3s encourages dispersal from feces and subsequent host seeking, while the attraction of free-living adults and non-infective larvae to host feces and fecal bacteria retains these life stages on host feces, where they grow and reproduce [27], [124]. Olfactory preferences in *S. ratti* can also vary depending on the cultivation temperature of the infective larvae [125]. Whether similar temperature-dependent olfactory plasticity occurs in *S. stercoralis* is unknown.

Remarkably little is known about the neural or molecular basis of olfaction in *Strongyloides* or any other parasitic nematode species. In *C. elegans*, the primary olfactory neurons are the AWA, AWB, and AWC neurons [109], [110]. The positional homologs of these neurons are candidate olfactory neurons in *Strongyloides,* but their functions have not yet been tested. As in mammals, the odorant receptors in *C. elegans* and other nematodes comprise a large family of seven-transmembrane domain, G protein-coupled receptors (GPCRs) [76], [109], [126], [127]. Members of the chemoreceptor gene family have been functionally characterized only in *C. elegans* [109], [128]. The chemoreceptor gene family is highly divergent across nematode species, likely reflecting species-specific olfactory requirements [126]. Moreover, the size of the chemoreceptor gene family in parasitic nematodes positively correlates with increased environmental exposure of the parasite [126]. For example, *S. stercoralis,* which has a free-living generation, has more chemoreceptors than *B. malayi*, a mosquito-borne filarial worm that is transmitted directly from host to vector to host [126], [127]. Expression of many *S. stercoralis* chemoreceptor genes is upregulated in iL3s relative to other life stages, consistent with an important role for chemosensation in host seeking and host invasion [75], [127].

In *C. elegans*, the odorant receptors act upstream of two different signal transduction pathways: a transient receptor potential (TRP) channel pathway that includes the TRP channel subunits OSM-9 and OCR-2, and a cGMP pathway that includes the cyclic nucleotide-gated (CNG) channel subunits TAX-2 and TAX-4 [109]. Upon ligand-mediated activation of the odorant receptors, G-protein signaling eventually leads to changes in neuronal activity via the TRP channel pathway in AWA neurons or the cGMP pathway in AWB and AWC neurons [109]. Some of the downstream effectors in these pathways are likely conserved across nematode species, and the functions of a few of these effectors have been characterized in parasitic nematodes [28], [29], [34], [126], [129]. In *S. stercoralis*, CRISPR/Cas9-mediated disruption of *Ss-tax-4* eliminates attraction to a host odorant [34]. Thus, cGMP signaling plays a conserved role in mediating chemosensory-driven host seeking in *S. stercoralis* and environmental navigation in *C. elegans*. The TRP channel pathway appears to play a role in chemoattraction in filarial nematodes [126], but whether it also mediates chemoattraction in *Strongyloides* spp. has not yet been assessed. Going forward, the expanding genetic toolkit for *Strongyloides* will facilitate the identification and functional characterization of neurons and molecular pathways required for olfactory behaviors in these parasites.

## 4 Gustatory behaviors of *Strongyloides* species

Gustation, or the detection of non-volatile chemicals, is thought to play a critical role in driving the host-seeking and host-invasion behaviors of *Strongyloides* iL3s. The behavioral responses of *Strongyloides* iL3s to sodium chloride (NaCl) are the most well-characterized, which is not surprising given that sodium and chloride are the most abundant electrolytes found in human sweat [130]. The behavioral responses to previously examined gustatory cues fall into three categories: chemotaxis, which describes the directed migration of animals in a chemical gradient; accumulation, which describes the retention of animals at a point source of the stimulus; and skin penetration.

When placed in an NaCl gradient, *S. stercoralis* iL3s migrate to ~30–70 mM NaCl [31], [32], a concentration range that overlaps that found in human sweat [131]. *S. stercoralis* iL3s also accumulate at point sources of NaCl in a similar concentration range [32]. The accumulation response of *S. stercoralis* iL3s is specific to NaCl since other chloride-containing compounds such as potassium chloride, calcium chloride, and magnesium chloride are not attractive to the iL3s [32]. Additionally, *S. stercoralis* iL3s accumulate at point sources of sodium hydroxide but not non-sodium-containing alkaline compounds, suggesting that sodium-sensitive receptors mediate the attractive response of *S. stercoralis* to NaCl [32]. These results raise the possibility that sodium ions also direct *S. stercoralis* migration within a human host, as sodium is the principal cation in the extracellular fluid [132].

In the case of *S. ratti*, iL3s accumulate near point sources of 50 mM NaCl [133]. When placed in an NaCl gradient at concentrations between 230 and 370 mM, *S. ratti* iL3s migrate down the gradient to concentrations under 80 mM [134]. However, whether *S. ratti* iL3s can migrate up NaCl gradients when placed at low NaCl concentrations remains unclear [134]. Together, these studies raise the possibility that *S. stercoralis* and *S. ratti* iL3s show species-specific gustatory responses, which need to be confirmed by more detailed, quantitative behavioral analyses.

The responses of *Strongyloides* iL3s to complex gustatory cues such as human sweat and sera have also been examined. *S. stercoralis* iL3s are attracted to human sweat and sera in an accumulation assay [32]. Whether they are attracted to components of these fluids other than NaCl is unknown [32]. Similar studies with *S. ratti* demonstrated that *S. ratti* iL3s are attracted to intact and dialyzed mammalian sera, as well as ovalbumin, albumins, and select peptides in accumulation assays [135]. However, the chemotaxis behaviors of *S. stercoralis* and *S. ratti* in response to these physiologically relevant gustatory cues have not yet been examined. Moreover, additional studies are required to assess whether skin and sweat compounds aside from NaCl –including lactate, urea, ammonia, lipids, and glycoproteins – also attract iL3s.

The role of gustatory cues in driving skin penetration remains poorly understood. *S. ratti* penetrates rat and mouse skin more readily than the skin of other taxonomically distant species (e.g., bird, dog, cat, and rabbit), raising the possibility that host-specific skin penetration relies upon species-specific gustatory cues that vary in chemical identity and/or concentration [136]. However, whether these differences in the rates of skin penetration are the result of differences in chemical cues or mechanical properties among skin types remains to be determined.

Current knowledge of the neural circuits associated with salt sensing in *C. elegans* has served as a foundation to understand salt sensing in *Strongyloides.* In *C. elegans*, the ASE neurons are the primary neurons that mediate attraction to gustatory cues, while the ASH neurons are the primary neurons that mediate repulsion [110]. *S. stercoralis* has positional homologs of the ASE and ASH neurons [69], and laser ablation of these neurons demonstrated that the ASE neurons mediate chemoattraction of iL3s to low concentrations of NaCl and the ASH neurons mediate chemorepulsion of iL3s from high concentrations of NaCl [117]. In future studies, newly developed techniques for chemogenetic silencing of individual neuron types and calcium imaging of neuronal activity in *Strongyloides* will be critical for further elucidating the neural circuitry that mediates salt sensing.

The molecular basis of salt sensing in *Strongyloides* has not yet been investigated. In *C. elegans*, gustatory responses are mediated by a large family of receptor guanylate cyclases (rGCs), almost half of which are expressed in ASE [109], [137]. Whether rGCs similarly act as gustatory receptors in *Strongyloides* has not yet been investigated. An in-depth characterization of the rGC family in *Strongyloides*, including the function of individual rGCs and their expression patterns, will provide important insights into the gustatory mechanisms that drive parasitic behaviors.

## 5 Carbon dioxide sensing in *Strongyloides* species

Carbon dioxide (CO_2_) gas is a critical cue for environmental navigation in numerous free-living and parasitic nematodes [138]. Consistent with their diverse lifestyles and ecological niches, responses to CO_2_ differ across nematode species [138]. In addition, there is also marked variation in CO_2_ response within species. Environmental context, recent experience, and developmental stage can all influence CO_2_-mediated behaviors. For example, iL3s of the passively ingested rodent parasite *Heligmosomoides polygyrus* are repelled by CO_2_ when isolated directly from feces but attracted to CO_2_ when removed from feces for multiple days [139]. The recent experience of the iL3s dictates their behavioral valence to an identical stimulus, allowing for plastic responses as environmental conditions change [139]. Plasticity of CO_2_-evoked behavior also occurs in *C. elegans*, where well-fed adults are repelled by CO_2_ [112], [140] while starved adults are attracted to CO_2_ [141].

*S. stercoralis* encounters variations in CO_2_ levels throughout its developmental cycle, with higher relative concentrations of CO_2_ in feces and within the host as compared to the ambient environment (~0.04 %) [142]. Early work demonstrated that exposure to human breath increased motility in *S. stercoralis* iL3s more than exposure to human breath from which the CO_2_ had been removed [33]; however, this movement was not assigned positive or negative valence. With the introduction of the CO_2_ chemotaxis assay, in which animal movement either toward or away from the gas stimulus is quantified, *S. stercoralis* and *S. ratti* iL3s demonstrated marked repulsion to CO_2_ [27]. This finding aligns with the expectation that iL3s must actively disperse away from feces during host seeking. In this setting, the high levels of CO_2_ present in feces may serve as a dispersal cue. Following departure from feces, iL3s navigate to the low CO_2_ environment of the host skin surface. Notably, *S. stercoralis* iL3s do not show flexible responses to CO_2_ depending on previously experienced environmental conditions; when removed from host feces – even for two weeks – *S. stercoralis* iL3s exhibit stable repulsion from CO_2_ [139].

CO_2_ also plays an important role in iL3 activation in *Strongyloides*. The standard procedure for in vitro activation of *S. stercoralis* iL3s involves incubating larvae in Dulbecco’s modified Eagle medium (DMEM) while maintaining a 37 °C and 5 % CO_2_ environment [34], [118], [127], [143]. Incubation at near-atmospheric CO_2_ levels, without other changes to the standard procedure, markedly hampers activation of *S. stercoralis* iL3s [34]. Thus, CO_2_ is a required cue for progression to the parasitic stages of the infectious life cycle.

Studies in *C. elegans* have provided useful insights into the neural and molecular mechanisms underlying CO_2_ responses in other nematodes. In *C. elegans*, the paired amphidal BAG neurons are the primary CO_2_-sensing neurons [112], [144], [145], although other neurons also contribute to CO_2_ detection [145], [146]. CO_2_ detection by BAG is mediated by the rGC GCY-9, a putative receptor for molecular CO_2_ [144], [147]. The BAG neurons play a conserved role in mediating CO_2_ response in the entomopathogenic nematodes *Heterorhabditis bacteriophora* and *Steinernema carpocapsae*, as well as the beetle-associated nematode *Pristionchus pacificus* [148]. However, the neural and molecular mechanisms of CO_2_ response in *Strongyloides* species, or any other mammalian-parasitic nematode species, remain to be elucidated.

## 6 Thermosensation

Many nematodes utilize thermotaxis to navigate the local environment [105], [149]. At physiological temperatures, *C. elegans* employs both positive and negative thermotaxis to navigate toward a preferred temperature range [111]. This temperature range mirrors the animals’ recent cultivation temperature (T_c_), allowing for plastic, experience-dependent tuning of temperature preferences. In addition, *C. elegans* adults avoid and escape from noxious heat stimuli (T > 26 °C) [150]. While skin-penetrating nematodes also exhibit experience-dependent thermotaxis, the exact behavioral responses diverge in support of parasitic host seeking.

*S. stercoralis* iL3s exposed to linear temperature gradients exhibit rapid movement toward host body temperature [28], [35]; this movement toward warmer temperatures in a thermal gradient is defined as positive thermotaxis. Conversely, negative thermotaxis is defined as movement toward cooler temperatures within a gradient. Long-range positive thermotaxis propels iL3s into proximity with a heat source, at which point chemosensory cues are suggested to provide more detailed information about the suitability of the putative host [28]. Worms are also able to course-correct, should the heat source ultimately be below host body temperature [29]. For example, *S. stercoralis* iL3s may home toward a sun-warmed rock, only to find that the environmental temperature gradient peaks at 25 °C. In a matter of minutes, worms that reach the perceived premature end of the temperature gradient reverse course and engage in negative thermotaxis toward cooler temperatures in the thermal gradient [29]. The aptitude for quick reversals allows iL3s to vacate “false positive” heat sources and resume searching for an appropriate host. This rapid behavioral adaptation of *S. stercoralis* is distinct from the adaptation of temperature preference in *C. elegans*, which emerges on the order of hours [151].

The temperatures at which *S. stercoralis* iL3s perform positive thermotaxis are dictated by the T_c_ of the worms [28]. When worms cultivated at 23 °C are placed at 25 °C or higher in a ~20–34 °C thermal gradient, most worms exhibit robust positive thermotaxis toward 34 °C. If placed at 22 °C or lower in the same thermal gradient, the worms instead move toward cooler temperatures. This negative thermotaxis behavior is posited to serve as a dispersal mechanism, potentially allowing for more precise detection of host heat amid a cooler ambient environment [28]. When iL3s are cultivated at 15 °C – even for only two hours prior to the assay – nearly all worms placed at 22 °C within a ~20–34 °C thermal gradient seek heat. Given that *S. stercoralis* iL3s are capable of rapidly shifting their temperature-driven behaviors as ambient temperature changes, it is plausible that parasites adhere to a crepuscular pattern of host seeking [28]. Currently available data support a model wherein dawn and dusk – times when ambient temperatures are cooler and humans are likely to be outside – are the prime times for host seeking [28], [105].

Beyond driving motility during host seeking, heat also serves as a critical developmental cue. In *S. stercoralis* and *S. ratti*, temperature directs the progression of female post-parasitic larvae into either the homogonic or heterogonic free-living cycle [116], [152]. At temperatures approaching 37 °C, *S. stercoralis* L1 larvae preferentially enter the homogonic cycle [116]. Heat, in this setting, may indicate that the local environment is too warm to support survival of free-living life stages or it may indicate the continued presence of a host. At temperatures below 34 °C, *S. stercoralis* preferentially employs the heterogonic cycle [116]. An inverse pattern is seen in *S. ratti*, as cooler temperatures promote entry into the homogonic cycle [152], [153]. Additionally, in vitro activation requires both 5 % CO_2_ and an ambient temperature of 37 °C [34]. Heat, alone, is insufficient to drive activation in *S. stercoralis* and *S. ratti*. However, when integrated with other host signals, the 37 °C environment is likely a reliable indicator of successful host invasion [34].

Given the relative similarities in nematode morphology and neuroanatomy, understanding of thermosensation machinery in *C. elegans* provides an excellent springboard for mechanistic study in *Strongyloides* spp. In *C. elegans*, a pair of amphid neurons in the head called the AFD neurons, named after their distinctive finger-like dendritic processes, are the primary sensors of temperature stimuli [111], [154], [155], [156], [157], [158], [159], [160]. *S. stercoralis* does not have sensory neurons with similar dendritic morphologies. Instead, the primary thermosensory neuron pair in *S. stercoralis* was called ALD, titled by the lamellar shape of the dendritic process [35]; this neuron was hypothesized to mediate thermosensation due, in part, to its increased dendritic surface area, a feature shared by *C. elegans* AFD [69], [115], [161]. *S. stercoralis* ALD was confirmed to play a role in thermotaxis behavior via laser ablation studies [35]. Following disruption of ALD, iL3s no longer exhibited directed movement toward a heat source [35]. Moreover, *S. stercoralis* worms with ablated ALD neurons did not display a temperature-dependent preference toward the homogonic cycle [116].

Recent advances in genetic and molecular tools have allowed for the reclassification of *Ss*-ALD as *Ss*-AFD, based on molecular identity [29]. In *C. elegans* AFD, the rGCs GCY-8, GCY-18 and GCY-23 are critical molecular components in mediating thermosensory response; at least two of these rGCs, GCY-18 and GCY-23, function directly as thermoreceptors [157], [160], [162], [163], [164]. These rGCs are specific to AFD and have been used as genetic markers for *C. elegans* AFD. *S. stercoralis* and *S. ratti* each have three homologs to *C. elegans* GCY-8, GCY-18, and GCY-23 [29]. These homologs phylogenetically cluster with *C. elegans* GCY-23, and have been named GCY-23.1, GCY-23.2, and GCY-23.3 in each *Strongyloides* species. Akin to the thermosensory rGCs in *C. elegans*, *S. stercoralis* GCY-23.1, GCY-23.2, and GCY-23.3 are expressed in a single pair of amphid neurons (Fig. 2A); by confocal microscopy, expression appears specific to the previously described *S. stercoralis* ALD neurons [29]. The *S. stercoralis* ALD neuron pair is hereafter referred to as *S. stercoralis* AFD, in reference to its molecular and functional characteristics.

Within *S. stercoralis*, the molecular machinery mediating the thermosensory response in AFD is still under investigation. The dynamic ranges, or spans of temperature over which rGCs can confer neural activity, of *S. stercoralis* GCY-23.1, GCY-23.2, and GCY-23.3 were explored by ectopically expressing each rGC in the *C. elegans* ASE chemosensory neurons. Within *C. elegans* ASE, all three *S. stercoralis* AFD-specific rGCs function as thermosensors and are capable of conferring a response range spanning host body temperature [29]. The native functions of GCY-23.1, GCY-23.2, and GCY-23.3 in *S. stercoralis* AFD have yet to be described. *C. elegans* GCY-8, GCY-18, and GCY-23 act upstream of a cGMP-gated cation channel encoded by the *tax-2* and *tax-4* genes [162], [165], [166]. The *S. stercoralis tax-4* homolog also plays a critical role in thermosensation. As demonstrated via CRISPR/Cas9-mediated deletion, *S. stercoralis tax-4* is required for thermotaxis [28], [29] (Fig. 2D). The *tax-4* gene is also required for the temperature-regulated process of activation in *S. stercoralis* [34].

Through the novel application of genetic tools in *S. stercoralis,* AFD has been confirmed as a thermosensory neuron with unique encoding properties and has been shown to play a critical role in thermotaxis in iL3s. Using the *Ss*-AFD-specific rGC promoters, targeted chemogenetic silencing and calcium imaging have illuminated the mechanistic role of *Ss*-AFD in mediating thermotaxis. When AFD is chemogenetically silenced by expression of HisCl1, *S. stercoralis* iL3s show diminished long-range thermotaxis behaviors [29] (Fig. 2B). Calcium imaging from AFD using the ratiometric calcium indicator yellow cameleon YC3.60 revealed that AFD responds to warming temperature ramps with a threshold temperature for neuronal response near ambient temperature. As the temperature rises above ambient, AFD displays an initial decrease in neural activity, followed by a near-linear increase as temperatures approach host body temperatures [29] (Fig. 2C). These response properties, apart from the threshold response, differ markedly from those in *C. elegans* AFD. The *C. elegans* AFD neurons display monotonic responses to heat only in a narrow temperature range adjacent to the recently experienced ambient temperature [29], [111], [158], [159], [160], [167]. The distinct neuronal encoding of *S. stercoralis* AFD enables the parasite to detect temperature changes in a range spanning from ambient to host body temperature. The persistent response of AFD across this temperature range likely mediates host-seeking behavior [29].

In integrating behavioral, molecular, and neuronal data, heat has been clearly demonstrated as a critical cue for *S. stercoralis* host seeking. Temperature also influences the developmental progression of various life cycle stages. Interventions aimed at disrupting thermosensation provide an exciting future avenue for nematode control. Future work is also needed to explicate the neural circuit connecting thermosensation to behavioral response.

## 7 Conclusion and future directions

Here, we have summarized key genetic and genomic tools adapted for use in *Strongyloides* spp. ─ including transgenesis, CRISPR/Cas9-mediated mutagenesis, chemogenetic neuronal silencing, and imaging with fluorescent biosensors ─ and illustrated the use of these tools in studying the sensory behaviors that drive host seeking and host infection in *Strongyloides*. In the future, the development of additional techniques to probe gene function will be essential for further studies of *Strongyloides* sensory neurobiology. For instance, the development of an auxin-inducible degradation (AID) system for *Strongyloides*, like that used in *C. elegans* [168], [169], would enable the conditional depletion of any endogenously tagged protein. An AID system would be especially useful for studying essential genes or genes that are required for host infection. The application of bipartite expression systems such as the Q- [170], Cre/Lox [171], or Split cGAL [172] systems to *Strongyloides* would also be invaluable for achieving temporal and/or spatial control of gene expression.

**Fig. 3:**
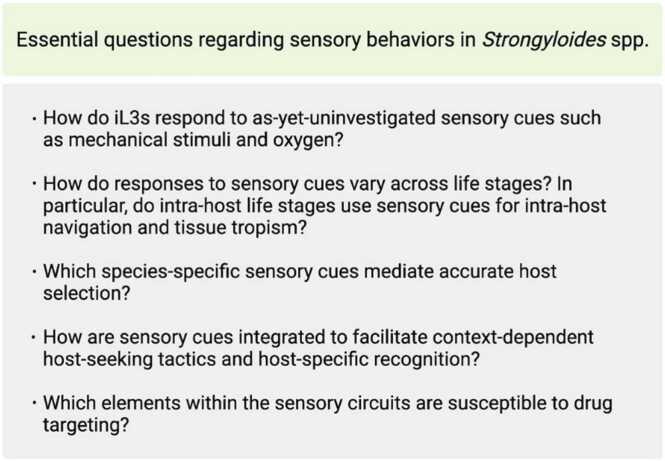
Essential questions regarding sensory behaviors in *Strongyloides* spp. With the current functional genetic and genomic toolkit for *Strongyloides*, the field is poised to address critical questions regarding host sensing and its underlying molecular and neural mechanisms in *Strongyloides*. A better understanding of the questions highlighted above will be critical for identifying essential parasitic behaviors required for host targeting, as well as neural and molecular components of host-sensing pathways that may be vulnerable to drug targeting.

Although the sensory behaviors of *Strongyloides* spp. are increasingly well-understood, many key questions remain to be addressed (Fig. 3). One key question is how *Strongyloides* spp. respond to additional sensory modalities. For example, oxygen is an important sensory cue for *C. elegans* and other free-living nematodes [113], [114], [173], [174], [175], but it remains unknown whether *Strongyloides* spp. sense oxygen. Similarly, mechanosensation is an important sensory modality for many nematodes, including *C. elegans* [176], entomopathogenic nematodes in the genera *Heterorhabditis* and *Steinernema* [177], [178], and the dog hookworm *Ancylostoma caninum* [179]. In future studies, it will be important to determine whether *Strongyloides* spp. utilize mechanosensory cues for host seeking and host invasion. Another important question is how sensory behaviors differ across life stages. Sensory behaviors have so far been studied primarily in infective larvae and free-living adults (Fig. 1). Looking forward, it will be necessary to understand how the parasitic life stages of *Strongyloides* use sensory cues for intra-host navigation. Additional investigation will also be necessary to pinpoint the species-specific sensory cues that mediate accurate host selection, or in the case of parasitic life stages, tissue tropism. How multiple sensory modalities are integrated to drive context-appropriate parasitic behaviors also remains to be investigated.

Finally, we still know remarkably little about the sensory neural circuits and sensory signaling pathways of *Strongyloides* or other parasitic nematodes. Although a few sensory neurons have been characterized in *Strongyloides*, nothing is currently known about the downstream interneurons and motor neurons that drive host-seeking and host-infection behaviors. Moreover, only a few molecular components required for host seeking have been identified. The identification of additional neurons and signaling components within these neurons could lead to the identification of novel drug targets. Taken together, a better understanding of sensory behaviors across life stages and ethologically relevant environmental contexts, as well as the neural circuits that underlie these behaviors, may drive the discovery of new approaches to preventing or treating nematode infections.

## CRediT authorship contribution statement

**Patricia Mendez:** Conceptualization, Writing – original draft, reviewing, and editing. **Breanna Walsh:** Conceptualization, Writing – original draft, Writing – review & editing. **Elissa Hallem:** Conceptualization, Supervision, Writing – review & editing.
